# Exploring the relationships between nutrition and brain health among Indigenous Peoples in North America: a systematic review

**DOI:** 10.17269/s41997-025-01078-6

**Published:** 2025-07-07

**Authors:** Anik Obomsawin, Joyla A. Furlano, Letebrhan Ferrow, Deyowidron’t Morrow, Guylaine Ferland, Laura E. Middleton, Lynden Crowshoe, Jennifer D. Walker, Alexandra J. Fiocco

**Affiliations:** 1 Psychology Department, Toronto Metropolitan University, Toronto, ON Canada; 2 Department of Family Medicine, Queen’s University, Kingston, ON Canada; 3 Department of Health Research Methods, Evidence, and Impact, Faculty of Health Sciences, McMaster University, Hamilton, ON Canada; 4 Indigenous Nutritional Knowledge Information Network, Dietitians of Canada, Toronto, Canada; 5 Department of Nutrition, Université de Montréal, Montreal, QC Canada; 6 Department of Kinesiology and Health Sciences, University of Waterloo, Waterloo, ON Canada; 7 Schlegel-UW Research Institute for Aging, Waterloo, ON Canada; 8 Department of Family Medicine, University of Calgary, Calgary, AB Canada

**Keywords:** Traditional Indigenous food systems, Indigenous population, Nutrition, Dietary contaminants, Brain health, Systèmes alimentaires traditionnels autochtones, Population autochtone, Nutrition, Contaminants alimentaires, Santé cérébrale

## Abstract

**Objectives:**

This systematic review synthesizes extant literature that examines relationships between nutrition and brain health in Indigenous populations in North America and further assesses the extent to which Indigenous research paradigms and community engagement processes have been employed.

**Methods:**

We searched five databases for primary research studies that examined indices of diet/nutrients in relation to brain health and focused on Indigenous populations in North America. Quality appraisal was performed using the Aboriginal and Torres Strait Islander Quality Appraisal Tool as well as the Appraisal Tool for Cross-Sectional Studies, the JBI Critical Appraisal Checklist for Case Control Studies, or the Critical Appraisal Skills Programme Cohort Study Checklist.

**Synthesis:**

Of the 564 articles identified in the search, 16 met inclusion criteria. Ten studies focused on Inuit populations, 2 focused on the residents of Grassy Narrows First Nation, 2 focused on Cree populations, and 2 focused on Quileute, Makah, and Quinault First Nations populations. Fourteen studies reported deleterious effects of dietary contaminants (e.g., mercury, lead, polychlorinated biphenyls) on brain health outcomes and three studies reported beneficial effects of omega-3 polyunsaturated fatty acids on brain health outcomes.

**Conclusion:**

Findings of this review highlight the neurotoxic effects of environmental contaminants and the beneficial effects of omega-3 polyunsaturated fatty acids in traditional Indigenous food sources on a range of brain health outcomes. However, given the methodological limitations of the studies reviewed and the lack of community-based research that employs Indigenous research paradigms, results should be interpreted with caution. There is a clear need for strength-based research that examines the positive effects of nutrients within traditional Indigenous foods on brain health.

**Supplementary information:**

The online version contains supplementary material available at 10.17269/s41997-025-01078-6.

## Résumé

**Objectifs:**

Cette revue systématique synthétise la littérature existante qui examine les associations entre la nutrition et la santé cérébrale des populations autochtones en Amérique du Nord, et évalue en outre la mesure dans laquelle les paradigmes de recherche autochtones et les processus d’engagement communautaire ont été utilisés.

**Méthodes:**

Nous avons recherché dans cinq bases de données des études de recherche primaire portant sur les indices de l’alimentation ou des nutriments en relation avec la santé cérébrale des populations autochtones en Amérique du Nord. L'évaluation de la qualité a été effectuée à l'aide de l'Aboriginal and Torres Strait Islander Quality Appraisal Tool ainsi que de l'Appraisal Tool for Cross-Sectional Studies, de la JBI Critical Appraisal Checklist for Case Control Studies ou de la Critical Appraisal Skills Programme Cohort Study Checklist.

**Synthèse:**

Sur les 564 articles identifiés lors de la recherche, 16 répondaient aux critères d'inclusion. Dix études portaient sur les populations Inuits, 2 sur les résidents de la Première Nation de Grassy Narrows, 2 sur les populations Cris, et 2 sur les populations des Premières Nations Quileute, Makah et Quinault. Quatorze études ont signalé des effets délétères des contaminants alimentaires (par exemple, mercure, plomb, biphényles polychlorés) sur la santé cérébrale et 3 études ont signalé des effets bénéfiques des acides gras polyinsaturés oméga-3 sur les résultats la santé cérébrale.

**Conclusion:**

Les résultats de cette revue mettent en évidence les effets neurotoxiques des contaminants environnementaux et les effets bénéfiques des acides gras polyinsaturés oméga-3 présents dans les aliments traditionnels autochtones, sur la santé cérébrale. Cependant, compte tenu des limites méthodologiques des études analysées et le manque de données qui utilisent les paradigmes de la recherche relative aux autochtones, les résultats doivent être interprétés avec prudence. De plus, cette revue souligne la nécessité de recherches qui examinent les effets positifs des nutriments contenus dans les aliments traditionnels autochtones, sur la santé cérébrale.

## Introduction

Indigenous Peoples around the world rely on traditional food^1^ systems, which are rooted in a deep understanding of the land, environment, and spiritual connection to nature (Kuhnlein & Chotiboriboon, 2022). These systems, informed by ancestral knowledge, have long guided the dietary practices of these populations. In Indigenous communities across North America, food sources often include wild and domesticated animals (e.g., moose, deer), fish local to their territories, and local edible plants/herbs and grains, all collected through sophisticated agricultural, hunting, fishing, harvesting, and gathering practices (Native Women’s Association of Canada, 2012; Park et al., 2016). Traditional Indigenous food practices, which vary between communities (Robin et al., 2021), promote physical well-being through access to food sources that are rich in micronutrients (Batal et al., 2021). Adhering to traditional Indigenous food patterns also promotes wholistic wellness by facilitating self-determination, cultural connectedness, and connection to ancestral knowledge (Dennis & Robin, 2020).

Indigenous Peoples often face unique nutritional health challenges as a result of colonization, which has created a profound shift in the dietary intakes of many Indigenous communities. Colonial processes such as residential schooling and displacement from traditional lands precipitated the loss of traditional Indigenous food systems and an increased reliance on often highly processed store-bought foods (Coté, 2016). Even when accessible, traditional Indigenous food sources are sometimes contaminated due to environmental changes such as habitat destruction, industrial pollution, and agricultural runoff, causing adverse health outcomes (Donaldson et al., 2010; Moriarity et al., 2021; Scheringer, 2009). Furthermore, many Indigenous families and communities suffer from food insecurity due to factors such as a lack of available traditional foods, a lack of available and affordable market foods in remote communities, economic disadvantages arising from structural violence, and government restrictions on hunting, fishing, and harvesting (Shafiee et al., 2022).

The loss of traditional Indigenous food systems and prevalence of food insecurity has been associated with an increase in adverse health outcomes among Indigenous populations (Coté, 2016). Indeed, Indigenous populations in Canada often experience a disproportionate prevalence of chronic illnesses that can be a result of malnutrition (e.g., undernutrition, inadequate vitamins or minerals; World Health Organization, 2024). For example, compared to non-Indigenous groups, Indigenous people experience a 12.2% and 2.1% higher prevalence of diabetes and cardiovascular disease, respectively, and 34% more dementia cases (Foulds et al., 2018; Jacklin et al., 2013; Crowshoe et al., 2018). Although food insecurity and increased consumption of Western diets have been associated with an increase in metabolic and cardiovascular diseases among Indigenous populations (Coté, 2016), the relative impact of dietary and nutrient intakes on brain health outcomes is under-investigated. Given that the aging population is steadily increasing, research on preventative and therapeutic strategies to maintain cognitive and brain health is critical, especially among high-risk groups.

The aim of this review is to synthesize current knowledge on the relationship between nutrition and indices of brain health in Indigenous populations in North America, and to assess whether appropriate Indigenous methodologies have been exercised in this research, including collaboration with communities. Research of this nature may help to promote and advance Indigenous self-determined research in this area to inform Indigenous dieticians and community health leaders in their planning and programming. The relationship between nutrition and wholistic wellness is complex and includes the revitalization of traditional Indigenous food systems grounded in ancestral knowledge, cultural reclamation, and self-determination of Indigenous Peoples (Coté, 2016; Dennis & Robin, 2020). Despite this complexity, for the sake of this review, nutrition is conceptualized as the intake of nutrients and/or contaminants through consumption of foods. This review aims to provide a springboard for future community-led research that may further this body of literature and account for sociocultural factors that impact the relationship between nutrition and brain health.

## Methods

### Statement of meaningful engagement with First Nations, Inuit, Métis, and Indigenous Peoples

Many authors on our team (Indigenous and non-Indigenous) have experience engaging in community-based research as well as research that employs Indigenous research paradigms, including the use of methods that align with Indigenous epistemologies, practicing relational accountability throughout the research process, and ensuring that Indigenous communities are engaged in and have control over research that concerns them. Through engagement with Indigenous and non-Indigenous communities, all authors have learned about ways in which culturally relevant foods and food practices promote wellness. These experiences have informed the conceptualization and methods of this review, as well as the interpretation of findings. Authors met throughout the research process to ensure that, despite the narrow scope of this review, findings were framed in such a way that drew attention to the importance of community-based research which employs Indigenous research paradigms and epistemologies. Central to working towards these objectives, the research team included Deyowidron’t Morrow, a Haudenosaunee registered dietitian from Six Nations of the Grand River. Deyowidron’t Morrow works to promote wellness in Indigenous communities through the cultural, spiritual, and physical benefits of community-specific foods and her involvement was integral in ensuring that findings of this review were contextualized by sociocultural factors that impact the relationship between nutrition and wholistic wellness.

### Overview

All stages of this systematic review, from protocol development to the reporting of results, were conducted in accordance with the Preferred Reporting Items for Systematic Reviews and Meta-Analyses (PRISMA) guidelines (Table S1; Page et al., 2021). A comprehensive search strategy was developed and implemented with the support of a librarian to identify relevant studies. Eligibility criteria, inclusion and exclusion processes, and data extraction methods were defined prior to beginning the review.

### Search strategy

Relevant literature published through January 18, 2023, were identified from CABI, OVID Medline, SCOPUS, CINAHL, and PsycINFO databases. Search terms included (diet* OR nutrition* OR food*) AND (cogniti* OR brain*) AND (Aboriginal* OR First Nation* OR Indigenous OR Inuit* OR Métis). A manual search of reference lists from articles included in the final review was also conducted. Two independent reviewers (A.O. and J.F.) assessed papers for inclusion at three assessment phases: assessment of title, assessment of abstract, and assessment of full paper. See supplementary material (Table S2) for a complete list of search terms and subject headings.

### Inclusion and exclusion criteria

Studies were included in the review if they (1) were full-text, primary research studies; (2) examined associations between indices of diet or nutrients and indices of brain health, including cognition and psychomotor/neuromotor function; and (3) focused on Indigenous populations in North America. Studies published in languages other than English were excluded. No exclusions were made based on forms of dietary or nutrient exposures.

### Data extraction

Data were independently extracted by A.O. and L.F. into a shared excel file that was subsequently reviewed for concordance. Data extracted included population of study, research objectives, research design, sample size, sex and age of sample participants, dietary or nutrient measure, brain or cognitive measure, dietary covariates considered in the analyses, and relevant results and effect size estimates (i.e., standardized regression coefficients, correlation coefficients, mean odds ratios, and differences in means), when available. Due to the heterogeneity in measures of diet and brain health, meta-analytic approaches were not employed.

### Assessment of methodological quality

To assess methodological quality of the included studies, four appraisal instruments were employed by A.O. and L.F. The Aboriginal and Torres Strait Islander Quality Appraisal Tool was adapted for a North American context and used to assess whether appropriate Indigenous research methodologies and community engagement were employed (Harfield et al., 2020). To assess study design, reporting quality, and risk of bias, one of three tools was used, depending on study design: the Appraisal Tool for Cross-Sectional Studies (AXIS tool, Downes et al., 2016), the JBI Critical Appraisal Checklist for Case Control Studies (Moola et al., 2017), or the Critical Appraisal Skills Programme (CASP) Cohort Study Checklist (CASP, 2018). The criteria list for each quality appraisal tool is included in supplementary material (Table S3). Studies that met a criterion received a score of 1; studies that did not meet a criterion received a score of 0; and studies that partially met a criterion received a score of 0.5. In cases where a criterion was not applicable, the criterion was removed from the list. The final quality assessment score was calculated as the percentage of criteria met for each study.

## Results

**Fig. 1:**
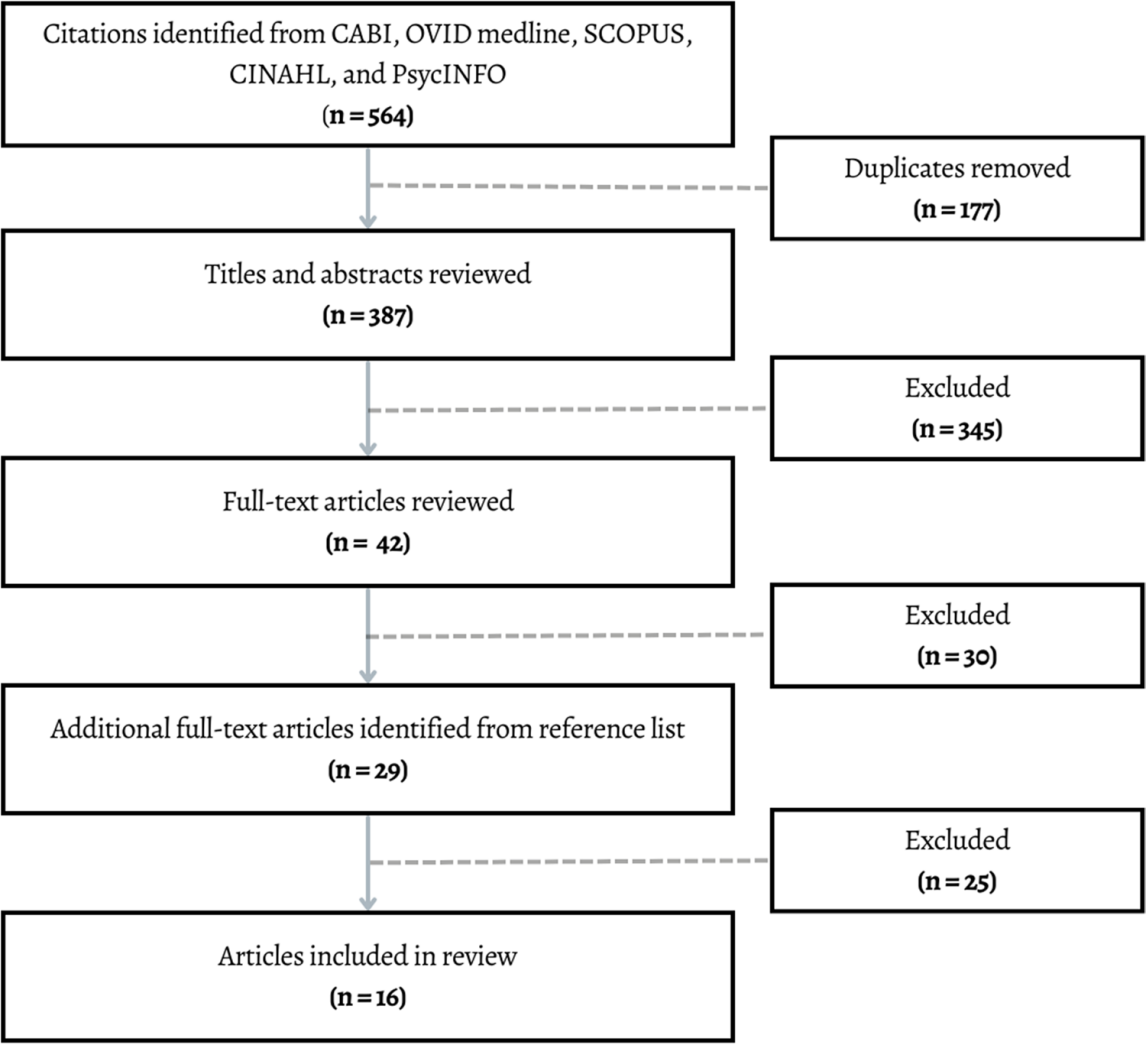
Flowchart summary of search results

A total of 564 articles were identified in the search. After removing duplicates, 387 articles were retained, of which 12 were included in the systematic review after the titles, abstracts, and full articles were reviewed. An additional 29 papers were extracted from the reference list of relevant papers, of which 4 were included in the systematic review. A list of studies that were included can be found in supplementary material (Table S4). In total, 55 studies were excluded after full-text review because they either did not include a brain health outcome (*k* = 33), were not primary studies (*k* = 10), did not include Indigenous populations (*k* = 8), were not North American (*k* = 3), or were inaccessible (*k* = 1). See Fig. 1 for the PRISMA flow diagram. A list of studies that were excluded after full-text review is included in supplementary material (Table S5).

### Population and study design

Of the 16 articles retrieved, 10 focused on Inuit populations, 2 focused on the residents of Grassy Narrows First Nation, 2 focused on Cree populations, and 2 focused on Quileute, Makah, and Quinault First Nations populations. Data were derived from 12 longitudinal studies, 2 cross-sectional studies, and 2 case–control studies. Of the 10 studies focusing on Inuit populations, 9 recruited participants from the Cord Blood Monitoring Program (1993–1998) which was initiated to document exposure to environmental contaminants and nutrients in newborns in Nunavik. Similarly, both studies in Quileute, Makah, and Quinault First Nations populations focused on The Communities Advancing the Studies of Tribal Nations Across the Lifespan (CoASTAL) cohort. Collectively, studies included 3737 participants, ranging in age from 11 months to over 83 years old. Characteristics of the 16 studies can be found in Table 1.

**Table 1:** Characteristics and summary of findings of 16 studies examining the relationship between diet and brain health in Indigenous populations. The following effect sizes were extracted when available: standardized regression coefficients ($\beta$), correlation coefficients (*r*), mean odds ratios (OR_mean_), and difference in means (*M*_diff_)

Authors and funders	Population	Objectives	Research design	Sample size, sex (%)	Mean age at recruitment (SD) or range, follow-up period (if applicable)	Dietary measures	Dietary confounding variables	Brain health outcomes	Summary of findings
Boucher et al. (2011) Funded by the NIH, the Northern Contaminants Program (INAC), Joseph Young Sr. Fund (State of Michigan), and CIHR	Inuit, Nunavik, Quebec, Canada	To examine the relation of *n* − 3 PUFAs and dietary contaminant intake with memory function in school age children	Longitudinal cohort	*N* = 154, 58.4% female	11.3 (0.6) years, 11 years	*n* − 3 PUFA, PCBs, and Hg in umbilical cord and child blood samples	Se and Pb levels in umbilical cord and child blood samples	Visual recognition memory and working memory via neuro-behavioural tests and event-related potentials during a visual recognition task	Children with higher prenatal *N* − 3 PUFA had event-related potentials indicative of better recognition memory compared to children with lower prenatal *n* − 3 PUFA (*M*_diff_ = 23.3***, − 2.9**) Prenatal *N* − 3 PUFA associated with better working memory ($\beta$= 0.35***) and recognition memory ($\beta$= 0.18*) in neuro-behavioural tests Children with higher current *n* − 3 PUFA had event-related potentials indicative of better recognition memory compared to children with lower current *N* − 3 PUFA (*M*_diff_ = 1.5*) Children with higher prenatal Hg had event-related potentials indicative of poorer recognition memory compared to children with lower prenatal Hg (effect sizes not unavailable) Prenatal Hg associated with poorer working memory ($\beta$= − 0.20*) in neuro-behavioural tasks Children with higher current PCB had event-related potentials indicative of poorer recognition memory compared to children with lower current PCB (effect sizes not unavailable) Child PCB associated with poorer working memory ($\beta$= − 0.18*) in neuro-behavioural tasks
Boucher et al. (2012) Funded by the NIH, the Northern Contaminants Program (INAC), and Joseph Young Sr. Fund (State of Michigan)	Inuit, Nunavik, Quebec, Canada	To examine response inhibition deficits associated with dietary contaminant intake in school age children	Longitudinal cohort	*N* = 196, 55.1% female	11.3 (0.6) years, 11 years	Pb, PCBs, and Hg levels in umbilical cord and child blood samples	*n* − 3 PUFA levels in umbilical cord and child blood samples	Response inhibition and executive control via event-related potentials during a visual response inhibition task	Child Pb ($\beta$= − 0.16*) and prenatal Pb ($\beta$= − 0.17*) associated with response inhibition impairment (i.e., increased impulsivity) Child PCB associated with error monitoring impairment ($\beta$= 0.18*) Prenatal PCB, prenatal Hg, and child Hg not associated with any measures
Boucher et al. (2014)^a^ Funded by the NIH, the Northern Contaminants Program (INAC), Joseph Young Sr. Fund (State of Michigan), Health Canada, and Hydro Quebec	Inuit, Nunavik, Quebec, Canada	To examine the effects of dietary contaminant intake on cognitive development in infants	Longitudinal cohort	*N* = 91, 36.2% female	50.5 (6.9) weeks, 11 months	PCBs, Hg, and Pb, in umbilical cord samples	Se, and *n* − 3 PUFA in umbilical cord samples	Recognition memory, information processing, working memory, and psychomotor development via neuro-behavioural tasks	Prenatal PCB associated with impaired recognition memory ($\beta$= − 0.17*) Prenatal Hg associated with impaired working memory ($\beta$= − 0.25*) Prenatal Pb associated with slower speed of processing ($\beta$= 0.21**) Contaminants not associated with mental and psychomotor development
Després et al. (2005) Funded by the Northern Contaminants Program (INAC), Health Canada, Hydro Quebec, and March of Dimes Birth Defect Foundation	Inuit, Nunavik, Quebec, Canada	To examine the effects of dietary contaminant intake on neuromotor development in preschool children	Longitudinal cohort	*N* = 110, 55.4% female	5.4 (0.4) years, 5 years	Hg, PCBs, Pb, OCPs, Se, *n* − 3 PUFA in umbilical cord and child blood samples	None	Neuromotor function (postural hand tremor, reaction time, standing postural sway oscillations, rapid pointing movements, and rapid alternating movements) via quantitative neuromotor tests	Neuromotor function was not linked to prenatal exposures Child Pb associated with changes in reaction time (*r* = 0.21*), sway oscillations (*r* = 0.24**), alternating arm movements (*r* = 0.24**), and action tremor (*r* = 0.32***) Child Hg associated with action tremor (*r* = 0.29**) PCBs, OCPs, *n* − 3 PUFA, and Se not associated with neuromotor development
Saint-Amour et al. (2006) Funded by the Northern Contaminants Program (INAC), Health Canada, Hydro Quebec, and March of Dimes Birth Defect Foundation	Inuit, Nunavik, Quebec, Canada	To examine the impact of dietary contaminant exposure on visual information processing in preschool children	Longitudinal cohort	*N* = 102, 56% female	5.4 (0.4) years, 5 years	PCBs, Hg, and *n* − 3 PUFA levels in umbilical cord and child blood samples	Pb levels in umbilical cord and child blood samples	visual information processing via visual evoked potential recordings	Child PCB ($\beta$= 2.50**) and Hg ($\beta$= − 3.94**, − 3.90***, − 3.26***, − 3.18***) and, to a lesser extent, prenatal Hg ($\beta$= 3.34*) associated with altered visual evoked potential responses consistent with subclinical impairments to visual information processing Child *n* − 3 PUFA associated with altered visual evoked potential responses consistent with improved visual information processing ($\beta$= − 5.71**) Neither *n* − 3 PUFA or Se offered protection against contaminants
Ethier et al. (2012) Funded by the Northern Contaminants Program (INAC), NIH, the Joseph Young Sr. fund (State of Michigan), CIHR, NSERC, FRSQ, Nasivvik Centre, and the Nunavik Regional Board of Health and Social Services	Inuit, Nunavik, Quebec, Canada	To examine the impact of dietary contaminant exposure on visual information processing in school age children	Longitudinal cohort	*N* = 149, 50% female	10.9 (0.6) years, 11 years	PCBs, Hg, and Pb levels in umbilical cord and child blood samples	Se and *n* − 3 PUFA levels in umbilical cord and child blood samples	Visual information processing via visual evoked potential recordings	Prenatal Hg ($\beta$= 0.21*) and Pb ($\beta$= 0.24*) associated with altered visual evoked potential responses at age 11 years consistent with subclinical impairments to visual information processing PCBs did not associate with altered visual evoked potential responses
Jacobson et al. (2008) Funded by the Northern Contaminants Program (INAC), NIH, Health Canada, Hydro Quebec, and the Joseph Young Sr. fund (State of Michigan)	Inuit, Nunavik, Quebec, Canada	To examine the relation between *n* − 3 PUFA intake on visual acuity and cognitive and motor development infants	Longitudinal cohort	*N* = 109, Sex not noted	Not specified, 11 months	*n* − 3 PUFA, in umbilical cord, and maternal blood and milk samples	PCBs, Hg, and Pb levels in umbilical cord samples	Visual acuity, recognition memory, information processing, and psychomotor development via neuro-behavioural tests	Prenatal *n* − 3 PUFA associated with better visual acuity ($\beta$= 0.31**) and recognition memory ($\beta$= 0.30**) at 6 months, and better mental ($\beta$= 0.23*) and psychomotor ($\beta$= 0.24*) performance at 11 months Post-natal *n* − 3 PUFA did not associate with any indicator of cognitive or motor development
Lamoureux-Tremblay et al. (2021) Funded by CIHR, NIH, the Northern Contaminants Program (INAC), and FRSQ	Inuit, Nunavik, Quebec, Canada	To examine the relation between dietary contaminant exposure and brain fear-circuitry in adolescents	Longitudinal cohort	*N* = 71, 56.3% female	18.3 (0.1) years, 16.2–21.6 years	Hg, Pb, and PCB levels in umbilical cord and adolescent blood samples	None	Brain activation patterns of fear learning and emotion regulation via magnetic resonance imaging during fear conditioning and extinction tasks	Adolescents with high prenatal PCB had altered brain activation during fear learning compared to adolescents with low prenatal PCB (effect sizes not available) Adolescents with high prenatal Hg and current Pb had altered brain activation during emotion regulation compared to adolescents with low prenatal Hg and current Pb (effect sizes not available)
Plusquellec et al. (2007)^a^ Funded by the NIH, the Northern Contaminants Program (INAC), Health Canada, FRSQ, and Hydro Quebec		To examine the relation between dietary contaminant exposure and behavioural and cognitive function in infants	Cross-sectional	*N* = 169, 37.9% female	352.1 (39.5) days	Pb levels in umbilical cord and maternal blood samples	PCB levels in umbilical cord samples	Visual attention, orientation and engagement via neuro-behavioural tests and behavioural observation	Prenatal Pb was not associated with any measures using neuro-behavioural tests Prenatal Pb associated with direct observational measures of infant attention ($\beta$= − 0.20*)
Cartier et al. (2014) Funded by the NIH, the Northern Contaminants Program (INAC), the Joseph Young Sr. fund (State of Michigan), CIHR, and the Nunavik Regional Board of Health and Social Services	Inuit, Nunavik, Quebec, Canada	To examine the relation between dietary contaminant exposure and visual information processing in school age children	Longitudinal cohort	*N* = 150, 50.7% female	11.3 (0.6) years, 11 years	OCP levels in umbilical cord and child blood samples	Pb and Se levels in umbilical cord samples, and Pb and Hg levels in child blood samples	Visual information processing via visual evoked potential recordings	Prenatal ($\beta$= 0.72*) and child ($\beta$= − 1.44**) OCP associated with altered visual evoked potential responses at age 11 years consistent with subclinical impairments to visual information processing
Philibert et al. (2022)^a^ Funded by Health Canada, CIHR, and the Government of Ontario	Grassy Narrows First Nations, Ontario, Canada	To cluster self-reported symptoms of nervous system dysfunction and examine their associations with dietary contaminant exposure in adults	Longitudinal cohort	*N* = 391, 54% female	18–50 + years, follow-up period not noted	Hg levels from umbilical cord, child blood, and hair samples, and fish consumption	None	Self-reported symptoms (clusters) of nervous system dysfunction (extrapyramidal impairment, sensory impairment, cranial nerve disturbances, gross motor impairment, and neuro-cognitive deficits)	Long-term Hg exposure indirectly and directly associated with higher symptom frequency for all the clusters of nervous system dysfunction (effect sizes not available)
Takaoka et al. (2014)^a^ Funded by JSPS	Grassy Narrows First Nations, Ontario, Canada	To assess the effects of dietary contaminants on symptoms of nervous system dysfunction in adult Grassy Narrow residents as compared to adult Japanese cases and controls	Case–control	*N* = 332 (80 Grassy Narrows residents, 88 Japanese-exposed residents, 164 Japanese controls), 57.2% female	52.3 (9.2) years	Fish consumption (proxy for Hg exposure)	None	Symptoms of nervous system dysfunction (sensory impairment, somatic pain, visual impairment, hearing impairment, tasting and smelling problems, incoordination of the extremities, and other movement impairment) via self-report and neurological examination	Prevalence of neurological complaints and neurological abnormalities of Grassy Narrow older adults (*r* = 0.71**, 0.71**, 0.79**) and younger adults (*r* = 0.45**, 0.71**, 0.82**) residents were similar to Japanese-exposed residents
Auger et al. (2005)^a^ No funding noted	Cree, Quebec, Canada	To examine whether dietary contaminant exposure is associated with the presence of nervous system dysfunction in adults	Cross-sectional	*N* = 302, 47.7% female	43.4 (15.6) years	Hg levels from hair and blood samples	None	Symptoms of nervous system dysfunction (sensory disturbance, incoordination, speech abnormalities, auditory disturbance, tremor, motor abnormalities, cranial nerves, reflexes, and cognitive changes) via neurological tests	Hair Hg levels associated with tremors in adults under 40 years old (OR_mean_ = 4.47) but with no other outcome
McKeown-Eyssen et al. (1983) Funded by the Government of Canada, the Government of Québec, and the Donner Canadian Foundation	Cree communities of Mistassini and Great Whale, Quebec, Canada	To examine whether dietary contaminant exposure is associated with the presence of nervous system dysfunction in adults	Case–control	*N* = 220 (41 cases, 179 controls), 56.8% female	53.6 (12.1) years	Hg levels from hair and blood samples	None	Symptoms of nervous system dysfunction (reduction of visual fields and presence of neurological disease) via examination by neurologists. Neurological disease was diagnosed if 1 or more of the following symptoms were present: nystagmus, tremor, sensory loss, astereognosis, and reduction in 2-point discrimination	In the community of Mistassini, Hg exposure associated with symptoms of nervous system dysfunction (women, OR_mean_ = 2.9**; men, OR_mean_ = 5.1**), but not in the community of Great Whale
Grattan et al. (2016)^a^ Funded by the NIH	Quileute, Makah, and Quinault First Nations, Pacific Northwest, USA	To examine the impacts of dietary contaminant exposure on memory in adults	Longitudinal cohort	*N* = 513, 59% female	36.3 (12.4) years, 4 years	Razor clam consumption (proxy for DA exposure)	None	Simple and complex attention and concentration, constructional praxis, verbal memory, psychomotor speed, dexterity, and cognitive flexibility via standard neuro-psychological test batteries	High razor clam consumers (15 or more per month) had subclinical decrements on verbal memory (*M*_diff_ = − 2.4*, − 3.3*) compared to non-consumers, with other cognitive functions unaffected
Tracy et al. (2016) Funded by the NIH	Quileute, Makah, and Quinault First Nations, Pacific Northwest, USA	To characterize dietary contaminant exposure and cognitive function in the cohort	Longitudinal cohort	*N* = 678, 51.9% female	Adults, 40.4 (14.7) years; children, 8.5 (1.5) years, 4 years	Razor clam consumption (proxy for DA exposure)	None	Simple and complex attention and concentration, constructional praxis, verbal memory, visual memory, psychomotor speed, dexterity, and cognitive flexibility via standard neuro-psychological test batteries	DA levels in this study were low and the cognitive outcomes for all participants were within normal range

^a^Studies examined outcomes not included in the table that were deemed unrelated to brain health

*Hg*; mercury, *Pb*; lead, *PCB*; polychlorinated biphenyl, *OCP*; organochlorine pesticides, *DA*; domoic acid, *n − 3 PUFA*; omega-3 polyunsaturated fatty acids, *Se*; selenium, *NIH*; National Institutes of Health, *INAC*; Indian and Northern Affairs Canada, *CIHR*; Canadian Institutes for Health Research, *NSERC*; Natural Sciences and Engineering Research Council of Canada, *FRSQ*; Fonds de la recherche en santé du Québec, *JSPS*; Japan Society for the Promotion of Science; *P < 0.05; **P < 0.01; ***P < 0.001

### Measures of dietary and nutrient intakes

A majority of the retrieved articles examined contaminants derived from dietary intake (*k* = 15), with fewer studies examining dietary nutrient intakes (*k* = 4). Dietary contaminants included heavy metals such as mercury and lead, industrially synthesized chlorine compounds such as polychlorinated biphenyls (PCBs) and organochlorine pesticides (OCPs), and a naturally occurring biotoxin known as domoic acid. Articles that reported on dietary nutrient intake focused on omega-3 polyunsaturated fatty acids (*n *− 3 PUFA). Among the retrieved articles, 12 examined prenatal contaminant/nutrient intake from umbilical cord samples; 3 examined contaminant/nutrient intake during infancy from blood or hair samples, or from maternal milk or blood samples; 7 examined contaminant/nutrient intake during childhood or adolescence from blood or hair samples; 3 examined contaminant/nutrient intake during adulthood from hair or blood samples; 3 examined consumption of fish or seafood as a proxy for dietary contaminant intake in adults. Refer to *Dietary Measures* in Table 1.

### Brain health indices

The retrieved articles assessed a range of brain health indices, including memory (*k* = 5), attention (*k* = 3), executive functioning (e.g., response inhibition, emotion regulation, cognitive flexibility, error monitoring and executive control; *k* = 4), visual and information processing (*k* = 5), visual acuity (*k* = 1), and psychomotor, neuromotor, and nervous system function (e.g., gross motor and sensory system function; *k* = 9). Refer to *Brain Health Indices* in Table 1.

### Quality appraisal

Scores on the Aboriginal and Torres Strait Islander Quality Appraisal Tool ranged from 0% to 53.6% with a mean of 9.7%. Six of the 16 studies referenced conversations and consultations with Indigenous community partners (e.g., local Councils, Elders, and Chiefs), but did not specify the roles of community partners or adherence to First Nations Governance Principles of OCAP® (The First Nations Information Governance Centre, 2021) or Inuit Qaujimajatuqangit (IQ). Furthermore, most articles mentioned the worldviews and history of Indigenous community partners, but this acknowledgement often did not affect the research paradigm. Scores on critical appraisal tools to evaluate study design ranged from 55 to 100% with a mean of 90.1%. Total quality appraisal scores for each study can be found in Table 1 and scores for each item/criterion of each quality appraisal tool are included in supplementary material (Tables S6-S9).

**Table 2:** Quality appraisal scores for 16 studies examining the relationship between diet and brain health in Indigenous populations

Authors	Aboriginal and Torres Strait Islander Quality Appraisal Tool Score (%)	Critical Appraisal Skills Programme (CASP) Cohort Study Checklist Score (%)	JBI Critical Appraisal Checklist for Case Control Studies Score (%)	Appraisal Tool for Cross-Sectional Studies (AXIS) Score (%)
Boucher et al. (2011)	3.6	100	—-	—-
Boucher et al. (2012)	0.0	100	—-	—-
Boucher et al. (2014)	7.1	87.5	—-	—-
Cartier et al. (2014)	7.1	100	—-	—-
Després et al. (2005)	7.1	100	—-	—-
Ethier et al. (2012)	7.1	91.7	—-	—-
Grattan et al. (2016)	3.6	100	—-	—-
Jacobson et al. (2008)	7.1	100	—-	—-
Lamoureux-Tremblay et al. (2021)	3.6	83.3	—-	—-
Philibert et al. (2022)	27.3	86.4	—-	—-
Saint-Amour et al. (2006)	7.1	100	—-	—-
Tracy et al. (2016)	53.6	95	—-	—-
McKeown-Eyssen et al. (1983)	7.1	—-	75	—-
Takaoka et al. (2014)	3.6	—-	55	—-
Auger et al. (2005)	3.6	—-	—-	80
Plusquellec et al. (2007)	7.1	—-	—-	87.5

### Key findings from studies

Fourteen studies reported deleterious effects of dietary contaminants on brain health outcomes and 3 studies reported beneficial effects of *n* − 3 PUFA on brain health outcomes. Key findings are summarized in Table 1.

#### Dietary contaminants

##### Mercury exposure

Ten studies examined mercury exposure among Inuit infants, children, and adolescents as well as Grassy Narrows First Nation and Cree adults (*n* = 1866). In Inuit infants, prenatal mercury exposure was associated with poorer working memory but not recognition memory (Boucher et al., 2014). However, in Inuit children, prenatal mercury exposure was associated with poorer recognition memory as well as working memory (Boucher et al., 2011). Prenatal mercury exposure was also associated with subclinical impairments to visual information processing among preschool (Saint-Amour et al., 2006) and school-aged (Ethier et al., 2012) Inuit children. Compared to prenatal mercury levels, mercury levels measured on the day of testing were more predictive of subclinical impairments to visual information processing in Ethier et al. (2012) but not in Saint-Amour et al. (2006). Current mercury levels were also associated with the presence of tremors in Inuit children (Després et al., 2005). Among Inuit adolescents, prenatal exposure to mercury was associated with altered brain activation during emotion regulation tasks (Lamoureux-Tremblay et al., 2021).

Among Grassy Narrows First Nation adults, mercury exposure was associated with nervous system dysfunction across studies. Long-term mercury exposure was associated with later-life coexisting symptoms of nervous system dysfunction, including extrapyramidal impairment (e.g., tremors and balance impairment), sensory impairment (e.g., numbness and tingling in upper and lower limbs), cranial nerve disturbances (e.g., loss of smell and taste), gross motor impairment (e.g., symptoms affecting walking), and neuro-cognitive deficits (e.g., memory impairment and speech disorder; Philibert et al., 2022). When examining the relative effect of mercury exposure on nervous system dysfunction in Grassy Narrows adults and Japanese adults, Takaoka et al. (2014) found that the prevalence of nervous system dysfunction among these two groups was similar. Common symptoms of nervous system dysfunction among exposed Grassy Narrow residents included tandem gait (i.e., heel to toe walking) abnormalities, somatosensory disturbances (i.e., ability to feel pain and touch), hearing impairments, and incoordination of the upper and lower extremities (Takaoka et al., 2014).

In Cree younger adults, Auger et al. (2005) found that mercury levels at the time of testing were associated with tremors but were not associated with other nervous system functions, including sensory disturbance, incoordination, speech abnormalities, auditory disturbance, motor abnormalities, cranial nerves, reflexes, and cognitive changes. McKeown-Eyssen et al. (1983) reported an association between mercury exposure and the presence of nervous system dysfunction (i.e., reduction of visual fields and presence of neurological disease) among adults in the Cree community of Mistassini. However, the nature of this dysfunction was not specified, and this association was not observed in the Cree community of Great Whale (McKeown-Eyssen et al., 1983).

##### Lead exposure

Six studies examined lead exposure among Inuit infants, children, and adolescents (*n* = 786). Among Inuit infants, prenatal lead exposure was associated with slower speed of information processing (Boucher et al., 2014). Prenatal lead exposure was also associated with adverse effects on observational measures of attention among Inuit infants but not with attention, orientation, or engagement measured via neuro-behavioural tests (Plusquellec et al., 2007). Among preschool-aged Inuit children, lead levels at the time of testing were associated with alterations to neuromotor function such as tremors, slower reaction time, greater postural sway, and less coherence between hands in alternating movements (Desprès et al., 2005). Among school-aged Inuit children, prenatal lead exposure was associated with subclinical visual information processing impairments (Ethier et al., 2012). In this population, lead levels at the time of testing were associated with response inhibition impairment (i.e., increased impulsivity; Boucher et al., 2012). Finally, among Inuit adolescents, current lead levels were associated with altered brain activation during emotion regulation tasks (Lamoureux-Tremblay et al., 2021).

##### Polychlorinated biphenyl and organochlorine pesticide exposure

Seven studies examined PCB exposure among Inuit infants, children, and adolescents (*n* = 873) and two studies examined OCP exposure among Inuit children (*n* = 260). In Inuit infants, prenatal exposure to PCBs was associated with impaired visual recognition memory (Boucher et al., 2014). Relatedly, in Inuit children, PCB levels at the time of testing were associated with poorer recognition, verbal short-term, and working memory (Boucher et al., 2011). Current PCB levels in school-aged Inuit children were also associated with altered brain activity involved in error monitoring (Boucher et al., 2012). Findings on the effects of PCB and OCP exposure on information processing in children were mixed. While pre- and postnatal OCP exposure was associated with subclinical visual information processing impairments among Inuit children (Cartier et al., 2014), Saint-Amour et al. (2006) found that only childhood PCB levels were associated with subclinical impairments to visual information processing and Ethier et al. (2012) found no significant effect of PCB exposure on visual information processing in Inuit children using the same methodology. Finally, among Inuit adolescents, prenatal exposure to PCBs was associated with altered prefrontal brain activation in emotion regulation tasks (Lamoureux-Tremblay et al., 2021).

##### Domoic acid exposure

Two studies investigating the impacts of domoic acid exposure through razor clam consumption (*n* = 1191) reported null associations with major domains of cognitive functioning (i.e., attention and concentration, the ability to manipulate spatial patterns, verbal memory, psychomotor speed, dexterity, and cognitive flexibility) among exposed children and adults from Quileute, Makah, and Quinault First Nations (Grattan et al., 2016; Tracy et al., 2016). Grattan et al. (2016) found that adults who consumed more razor clams had reduced verbal memory compared to adults who consumed lesser amounts; however, the relatively low verbal memory scores were not clinically meaningful.

#### Omega-3 polyunsaturated fatty acids

Prenatal exposure to *n* − 3 PUFA was associated with better visual acuity and recognition memory in 6-month-old Inuit infants, and better performance on mental and psychomotor tasks in 11-month-old Inuit infants (Jacobson et al., 2008). Saint-Amour et al. (2006) found that childhood intake of *n* − 3 PUFA improved visual information processing among preschool Inuit children. Regardless of exposure to seafood contaminants, prenatal *N* − 3 PUFA levels were associated with better verbal short-term memory, working memory, visual information processing, and enhanced brain activity during the recollection recall of visual information among school-aged Inuit children (Boucher et al., 2011).

## Discussion

With a growing body of research highlighting the role of a healthy diet for optimal brain health, this systematic review aimed to synthesize existing research on the relationship between nutrition and indices of brain health in North American Indigenous populations. Notably, a majority of the studies focused their investigation on food contaminants that stem from environmental toxins, including mercury, lead, PCBs, and OCPs. Together, these studies provide evidence for the detrimental effect of these dietary contaminants on emotion regulation processing, executive functioning, memory, and motor function among Indigenous populations in North America, notably within the Inuit population. Although fewer studies focused their investigation on brain-healthy nutrients, consumption of *n* − 3 PUFAs, which are often abundant in traditional Indigenous foods (e.g., cold water fish; Batal et al., 2021), was found to associate with improved visual information processing and acuity, visual recognition memory, verbal short-term memory, working memory, and mental and psychomotor performance.

Findings of this review align with previous literature on the effects of contaminants and *n* − 3 PUFA consumption on brain health (e.g., O’Connor et al., 2001; Iqubal et al., 2020; Willates et al., 1998; Witte et al., 2014). Mercury, lead, PCBs, and OCPs are widespread environmental contaminants found in the waters and lands of Indigenous communities (Donaldson et al., 2010; Moriarity et al., 2021; Scheringer, 2009), and exposure to these contaminants has been associated with a range of neurological abnormalities across populations (Iqubal et al., 2020). In line with findings of this review, most neurological abnormalities investigated include memory loss, visual impairments, and motor dysfunction (Azevedo et al., 2012; Puertas et al., 2010; Ribas-Fitó et al., 2003; Sanders et al., 2009; Zoeller et al., 2002). Furthermore, prenatal exposure to environmental contaminants is thought to be especially deleterious to the developing brain and nervous system (World Health Organization, 2007; Zoeller et al., 2002). Conversely, *n* − 3 PUFAs may improve neurological function. Indeed, prior studies have shown that consumption of *n* − 3 PUFAs promotes visual acuity and functioning, problem solving skills, cognitive development, and prevention of neurodegeneration (O’Connor et al., 2001; Willates et al., 1998; Witte et al., 2014).

Overall, the findings of this review do not capture the beneficial effects of traditional Indigenous diets on brain health. Most studies failed to collect information on dietary nutrient intakes (*k* = 8) or treated these variables as covariates (*k* = 4) without examining their unique contribution to the study outcome of interest. This is surprising given the widely acknowledged health benefits of traditional Indigenous foods. Indeed, many traditional Indigenous diets are found to be an excellent source of important macronutrients, including essential amino acids and fatty acids; micronutrients, including vitamins D, A, E, and B6; and minerals, including iron, copper, and zinc, (Batal et al., 2021). Many of these nutrients have been shown to play an important role in supporting optimal brain health across populations, especially polyunsaturated fatty acids (van Gelder et al., 2007); vitamins D, E, and B6 (Bryan et al., 2002; Perkins et al., 1999; Przybelski & Binkley, 2007); zinc (Ortega et al., 1997); selenium (Gao et al., 2007); copper (Pajonk et al., 2005); and iron (Murray-Kolb & Beard, 2007). Together, these findings suggest a variety of advantages associated with consuming traditional Indigenous foods, though studies examining this supposition were not identified in this review.

The maintenance and revitalization of community-specific food systems is widely acknowledged as a priority across Indigenous communities in North America (Coté, 2016). Rooted in ancestral knowledge, connection to community-specific food systems fosters cultural and spiritual relationships with the land which have been disrupted by colonialism (Martens et al., 2016). Renewing relationships to the natural world in turn strengthens Indigenous languages, cultural teachings, and family and community connections (Corntassel, 2008). The food sovereignty movement also helps to shape healthy and sustainable communities by increasing self-determination and decreasing dependence upon colonial and globalized food systems (Coté, 2016). From fighting for fishing rights in Mi’kma’ki to establishing Indigenous-led farming initiatives in Kitwanga, Indigenous groups across North America are reclaiming their lands and food systems.

This research, however, highlights the deleterious effects and pervasiveness of environmental contaminants in traditional Indigenous food sources. Indeed, Inuit from Nunavik are one of the most heavily exposed populations in the world to environmental contaminants due to bioaccumulation in fish and other seafood on which Inuit people rely (Muckle et al., 2001). Grassy Narrows First Nations communities in Northwestern Ontario still suffer from the aftermath of 10,000 kg of mercury being discharged into a nearby river system in the 1960 s (Rudd et al., 1983). Furthermore, decades of industrial development and long-range transport has resulted in widespread environmental contamination across North American Indigenous communities (Donaldson et al., 2010; Moriarity et al., 2021; Scheringer, 2009). Given the importance of traditional Indigenous food systems and the interconnectedness between Indigenous Peoples and their ancestral lands, it is imperative that there are policies in place to reduce the release of contaminants into the environment from anthropogenic activities such as metal mining, coal-fired power generation, and waste incineration (Government of Canada, 2019). In collaboration with Indigenous communities, risk assessment research which monitors the concentrations of contaminants in traditional Indigenous foods as well as the health implications of environmental contaminants over the lifespan should continuously be conducted. Furthermore, there should be an emphasis upon clear communication of risks as well as benefits of consumption of traditional Indigenous foods, and involvement of Indigenous communities at all stages of the risk management and communication process (Donaldson et al., 2010).

Notably, findings from the Aboriginal and Torres Strait Islander Quality Appraisal Tool indicated that almost all studies failed to employ appropriate Indigenous research methodologies and community engagement. For example, none of the studies noted Indigenous Peoples’ ownership or governance of the data and only one study acknowledged Indigenous research leadership and benefit to the Indigenous community (Tracy et al., 2016). Although most studies fared relatively well with respect to the quality of the study design using relevant quality appraisal tools, important methodological limitations of the studies included in this review must be addressed. For example, some studies were limited by small sample sizes or sample attrition (e.g., Boucher et al., 2014; Lamoureux-Tremblay et al., 2021; Philibert et al., 2022), which may increase the risk for type I and type 2 error. Furthermore, the use of instruments that are not validated in Indigenous populations, namely the emotion regulation task employed by Lamoureux-Tremblay et al. (2021), may have resulted in measurement error. Issues of confounding may also warrant cautious interpretation of the results. For example, in Takaoka et al. (2014), nervous system dysfunction in Grassy Narrows adults was compared to Japanese adults with very different lifestyles and confounding variables that were not identified or accounted for. Similarly, while McKeown-Eyssen et al. (1983) reported an association between mercury exposure and the presence of nervous system dysfunction among adults in the Cree community of Mistassini, there were differences between cases and controls in this study (e.g., cases were older and reported drinking more alcohol) and although the measured confounding variables were statistically accounted for, extraneous variables associated with group differences may account for the observed effects. As such, the association between mercury exposure and nervous system dysfunction among Grassy Narrows First Nation and Cree adults should be further examined.

Although a comprehensive search strategy was developed and implemented, the current systematic review may have been limited by the chosen Boolean search. Specifically, “Native” was not included within the Boolean terms as it yielded a disproportionate number of references on plant and animal species. As such, it is possible that articles using the term “Native” were excluded from this review, potentially limiting representation of studies conducted in the United States. Nonetheless, as the first systematic review of research on nutrition and brain health in North American Indigenous populations, it is clear that additional research is warranted. There is a clear dearth of strengths-based research that examines the benefits of consuming traditional Indigenous foods on brain health. Such research is needed to understand the extent to which the benefits of traditional Indigenous foods offset the risks of environmental contaminants and may also have implications for addressing the disproportionate rates of dementia in Indigenous communities (Warren et al., 2015). Furthermore, within the body of literature focusing on the relationships between diet and brain health, there is an inadequate representation of Indigenous communities across North America and lack of research examining this relationship across the lifespan. Among the 16 studies identified, 10 studies were conducted with Inuit populations and 10 studies focused only on infants and children. Future research should aim to examine the long-term risks and benefits associated with community-specific food systems across Indigenous populations. Finally, as highlighted by the quality appraisal process, there is a lack of community-based research which employs Indigenous research paradigms and epistemologies. This approach is essential to produce knowledge that responds to community priorities; incorporates mental, physical, emotional, and spiritual dimensions of health and wellness; and accounts for sociocultural factors involved in the use of traditional Indigenous food systems.

## Supplementary information

Below is the link to the electronic supplementary material.

ESM 1 (DOCX 47.0 KB)

## Data Availability

Not applicable.

## Footnotes

1 Throughout this paper, authors use the terms “traditional food” or “traditional Indigenous food” to describe foods, food practices, and food systems that are specific to a particular Indigenous community. However, it is important to note that this terminology may vary across Indigenous communities.
