# Supplementary material for: Exploring the relationships between nutrition and brain health among Indigenous Peoples in North America: a systematic review
Source: Can J Public Health. 2025 Jul 7;117(2):261–79. doi: 10.17269/s41997-025-01078-6 (PMC13076735; doi:10.17269/s41997-025-01078-6)
Supplement: Supplementary file 1 — (DOCX 47.0 KB) [file 41997_2025_1078_MOESM1_ESM.docx]

**Exploring the relationships between nutrition and brain health among Indigenous Peoples in North America: A systematic review**

**Supplementary Material**

**Table S1.** The Preferred Reporting Items for Systematic Reviews and Meta-Analyses (PRISMA) checklist.

| **Section and Topic** | **Item** | **Checklist item** |
| --- | --- | --- |
| **TITLE** | | |
| Title | 1 | Identify the report as a systematic review. |
| **ABSTRACT** | | |
| Abstract | 2 | Include an abstract summarizing objectives, methods, results, and conclusions. |
| **INTRODUCTION** | | |
| Rationale | 3 | Describe the rationale for the review in the context of existing knowledge. |
| Objectives | 4 | Provide an explicit statement of the objective(s) or question(s) the review addresses. |
| **METHODS** | | |
| Eligibility criteria | 5 | Specify the inclusion and exclusion criteria for the review and how studies were grouped for the syntheses. |
| Information sources | 6 | Specify all databases, registers, websites, organisations, reference lists and other sources searched or consulted to identify studies. Specify the date when each source was last searched or consulted. |
| Search strategy | 7 | Present the full search strategies for all databases, registers and websites, including any filters and limits used. |
| Selection process | 8 | Specify the methods used to decide whether a study met the inclusion criteria of the review, including how many reviewers screened each record and each report retrieved, whether they worked independently, and if applicable, details of automation tools used in the process. |
| Data collection process | 9 | Specify the methods used to collect data from reports, including how many reviewers collected data from each report, whether they worked independently, any processes for obtaining or confirming data from study investigators, and if applicable, details of automation tools used in the process. |
| Data items | 10a | List and define all outcomes for which data were sought. Specify whether all results that were compatible with each outcome domain in each study were sought (e.g. for all measures, time points, analyses), and if not, the methods used to decide which results to collect. |
|  | 10b | List and define all other variables for which data were sought (e.g. participant and intervention characteristics, funding sources). Describe any assumptions made about any missing or unclear information. |
| Study risk of bias assessment | 11 | Specify the methods used to assess risk of bias in the included studies, including details of the tool(s) used, how many reviewers assessed each study and whether they worked independently, and if applicable, details of automation tools used in the process. |
| Effect measures | 12 | Specify for each outcome the effect measure(s) (e.g. risk ratio, mean difference) used in the synthesis or presentation of results. |
| Synthesis methods | 13a | Describe the processes used to decide which studies were eligible for each synthesis (e.g. tabulating the study intervention characteristics and comparing against the planned groups for each synthesis (item #5)). |
|  | 13b | Describe any methods required to prepare the data for presentation or synthesis, such as handling of missing summary statistics, or data conversions. |
|  | 13c | Describe any methods used to tabulate or visually display results of individual studies and syntheses. |
|  | 13d | Describe any methods used to synthesize results and provide a rationale for the choice(s). If meta-analysis was performed, describe the model(s), method(s) to identify the presence and extent of statistical heterogeneity, and software package(s) used. |
|  | 13e | Describe any methods used to explore possible causes of heterogeneity among study results (e.g. subgroup analysis, meta-regression). |
|  | 13f | Describe any sensitivity analyses conducted to assess robustness of the synthesized results. |
| Reporting bias assessment | 14 | Describe any methods used to assess risk of bias due to missing results in a synthesis (arising from reporting biases). |
| Certainty assessment | 15 | Describe any methods used to assess certainty (or confidence) in the body of evidence for an outcome. |
| **RESULTS** | | |
| Study selection | 16a | Describe the results of the search and selection process, from the number of records identified in the search to the number of studies included in the review, ideally using a flow diagram. |
|  | 16b | Cite studies that might appear to meet the inclusion criteria, but which were excluded, and explain why they were excluded. |
| Study characteristics | 17 | Cite each included study and present its characteristics. |
| Risk of bias in studies | 18 | Present assessments of risk of bias for each included study. |
| Results of individual studies | 19 | For all outcomes, present, for each study: (a) summary statistics for each group (where appropriate) and (b) an effect estimate and its precision (e.g. confidence/credible interval), ideally using structured tables or plots. |
| Results of syntheses | 20a | For each synthesis, briefly summarise the characteristics and risk of bias among contributing studies. |
|  | 20b | Present results of all statistical syntheses conducted. If meta-analysis was done, present for each the summary estimate and its precision (e.g. confidence/credible interval) and measures of statistical heterogeneity. If comparing groups, describe the direction of the effect. |
|  | 20c | Present results of all investigations of possible causes of heterogeneity among study results. |
|  | 20d | Present results of all sensitivity analyses conducted to assess the robustness of the synthesized results. |
| Reporting biases | 21 | Present assessments of risk of bias due to missing results (arising from reporting biases) for each synthesis assessed. |
| Certainty of evidence | 22 | Present assessments of certainty (or confidence) in the body of evidence for each outcome assessed. |
| **DISCUSSION** | | |
| Discussion | 23a | Provide a general interpretation of the results in the context of other evidence. |
|  | 23b | Discuss any limitations of the evidence included in the review. |
|  | 23c | Discuss any limitations of the review processes used. |
|  | 23d | Discuss implications of the results for practice, policy, and future research. |
| **OTHER INFORMATION** | | |
| Registration and protocol | 24a | Provide registration information for the review, including register name and registration number, or state that the review was not registered. |
|  | 24b | Indicate where the review protocol can be accessed, or state that a protocol was not prepared. |
|  | 24c | Describe and explain any amendments to information provided at registration or in the protocol. |
| Support | 25 | Describe sources of financial or non-financial support for the review, and the role of the funders or sponsors in the review. |
| Competing interests | 26 | Declare any competing interests of review authors. |
| Availability of data, code and other materials | 27 | Report which of the following are publicly available and where they can be found: template data collection forms; data extracted from included studies; data used for all analyses; analytic code; any other materials used in the review. |

**Table S2.** Search terms and subject headings used in literature searches to identify relevant studies from each database.

| **Journal** | **Theme** | **Keyword** | **Subject Headings** | **Extraction Date** |
| --- | --- | --- | --- | --- |
| CINAHL | Food/Nutrition | Diet* | (MH "Diet+") OR "Diet*" | July 9, 2022 |
|  |  | Nutrition* | (MH "Nutrition+") OR "nutrition*" OR (MH "Nutrition Services") |  |
|  |  | Food* | (MH "Food+") OR (MH "Access to Healthy Foods") OR "Food*" |  |
|  | Brain Health/Cognition | Cogniti* | (MH "Cognition+") OR (MH "Cognition Disorders") OR (MH "Cognitive Aging") OR (MH "Cognitive Dissonance") OR (MH "Cognitive Flexibility") OR "Cogniti*" |  |
|  |  | Brain* | (MH "Brain+") OR (MH "Hypoxia-Ischemia, Brain") OR (MH "Hypoxia, Brain") OR (MH "Large-Scale Brain Networks") OR (MH "Brain Neoplasms") OR (MH "Intracranial Hemorrhage") OR (MH "Intracranial Arterial Diseases") OR (MH "Brain Hemispheres") OR (MH "Dementia") OR (MH "Pick Disease of the Brain") OR "Brain*" |  |
|  | Indigenous | Aboriginal* | (MH "Indigenous Health") OR (MH "Aboriginal Canadians") OR (MH "Native Americans") OR "Aboriginal*" |  |
|  |  | First Nation* | (MH "First Nations of Canada") OR "First Nation*" |  |
|  |  | Indigenous | (MH "Indigenous Peoples+") OR (MH "Health Services, Indigenous") OR (MH "Traditional Healers") OR "Indigenous" |  |
|  |  | Métis | Métis | January 18, 2023 |
|  |  | Inuit* | (MH "Inuit") OR "Inuit*" |  |
| OVID Medline | Food/Nutrition | Diet* | exp Diet/ or Diet*.mp. or Diet, Healthy/ | June 20, 2022 |
|  |  | Nutrition* | nutrition*.mp. or exp Nutrition Therapy/ |  |
|  |  | Food* | food safety/ or food contamination/ or exp Food/ or exp Food Insecurity/ or Food*.mp. or exp Food-Drug Interactions/ or exp Food Ingredients/ or exp Food Security/ |  |
|  | Brain Health/Cognition | Cogniti* | cognition/ or exp cognitive dissonance/ or exp cognitive reserve/ or exp comprehension/ or exp metacognition/ or cognition disorders/ or auditory perceptual disorders/ or Cognitive Dysfunction/ or Cogniti*.mp. |  |
|  |  | Brain* | Brain diseases/ or amnesia, transient global/ or basal ganglia diseases/ or brain diseases, metabolic/ or brain neoplasms/ or cerebellar diseases/ or cerebrovascular disorders/ or encephalomalacia/ or epilepsy/ or hypothalamic diseases/ or hypoxia, brain/ or intracranial hypertension/ or intracranial hypotension/ or leukoencephalopathies/ or neuroaxonal dystrophies/ or thalamic diseases/ or Fornix, Brain/ or Hypoxia-Ischemia, Brain/ or Brain Damage, Chronic/ or Evoked Potentials, Auditory, Brain Stem/ or exp Brain/ or Brain Injury, Chronic/ or Brain Stem Infarctions/ or Brain Ischemia/ or Brain Waves/ or Brain Stem Neoplasms/ or Brain*.mp. |  |
|  | Indigenous | Aboriginal* | exp Indians, North American/ or aboriginal*.mp. |  |
|  |  | First Nation* | “First Nation*”.mp. |  |
|  |  | Indigenous | Indigenous Peoples/ or Health Services, Indigenous/ or Indigenous.mp. |  |
|  |  | Métis | metis.mp. or Indigenous Canadians/ | January 18, 2023 |
|  |  | Inuit* | exp *Inuits/ |  |
| PsychInfo | Food/Nutrition | Diet* | — | June 27, 2022 |
|  |  | Nutrition* | — |  |
|  |  | Food* | — |  |
|  | Brain Health/Cognition | Cogniti* | — |  |
|  |  | Brain* | — |  |
|  | Indigenous | Aboriginal* | — |  |
|  |  | First Nation* | — |  |
|  |  | Indigenous | — |  |
|  |  | Métis | — | January 18, 2023 |
|  |  | Inuit* | — |  |
| CABI | Food/Nutrition | Diet* | — | June 27, 2022 |
|  |  | Nutrition* | — |  |
|  |  | Food* | — |  |
|  | Brain Health/Cognition | Cogniti* | — |  |
|  |  | Brain* | — |  |
|  | Indigenous | Aboriginal* | — |  |
|  |  | First Nation* | — |  |
|  |  | Indigenous | — |  |
|  |  | Métis | — | January 18, 2023 |
|  |  | Inuit* | — |  |
| SCOPUS | Food/Nutrition | Diet* | — | June 20, 2022 |
|  |  | Nutrition* | — |  |
|  |  | Food* | — |  |
|  | Brain Health/Cognition | Cogniti* | — |  |
|  |  | Brain* | — |  |
|  | Indigenous | Aboriginal* | — |  |
|  |  | First Nation* | — |  |
|  |  | Indigenous | — |  |
|  |  | Métis | — | January 18, 2023 |
|  |  | Inuit* | — |  |

*Note: Literature searches within PsychInfo, CABI, and SCOPUS databases did not include any subject headings.*

**Table S3.** Criteria list for (1) the Aboriginal and Torres Strait Islander Quality Appraisal Tool; (2) the Appraisal Tool for Cross-Sectional Studies (AXIS); (3) the JBI Critical Appraisal Checklist for Case Control Studies; (4) the Critical Appraisal Skills Programme (CASP) Cohort Study Checklist.

| **Quality Appraisal Tool** | **Criteria/Question** |
| --- | --- |
| Aboriginal and Torres Strait Islander Quality Appraisal Tool | Did the research respond to a need or priority determined by the community? |
|  | Was community consultation and engagement appropriately inclusive? |
|  | Did the research have Indigenous research leadership? |
|  | Did the research have Indigenous governance? |
|  | Were local community protocols respected and followed? |
|  | Did the researchers negotiate agreements in regards to rights of access to Indigenous Peoples’ existing intellectual and cultural property? ^a^ |
|  | Did the researchers negotiate agreements to protect Indigenous Peoples' ownership of intellectual and cultural property created through the research? ^a^ |
|  | Did Indigenous Peoples and communities have control over the collection and management of research materials? ^a^ |
|  | Was the research guided by an Indigenous research paradigm? ^b^ |
|  | Does the research take a strengths-based approach, acknowledging and moving beyond practices that have harmed Indigenous Peoples in the past? |
|  | Did the researchers plan and translate the findings into sustainable changes in policy and/or practice? |
|  | Did the research benefit the community? |
|  | Did the research demonstrate capacity strengthening for Indigenous individuals? |
|  | Did everyone involved in the research have opportunities to learn from each other? |
| Appraisal Tool for Cross-Sectional Studies (AXIS tool) | Were the aims/objectives of the study clear? |
|  | Was the study design appropriate for the stated aim(s)? |
|  | Was the sample size justified? |
|  | Was the target/reference population clearly defined? (Is it clear who the research was about?) |
|  | Was the sample frame taken from an appropriate population base so that it closely represented the target/reference population under investigation? |
|  | Was the selection process likely to select subjects/participants that were representative of the target/reference population under investigation? |
|  | Were measures undertaken to address and categorize non-responders? |
|  | Does the response rate raise concerns about non-response bias? |
|  | If appropriate, was information about non-responders described? |
|  | Were dietary factors and outcome variables measured appropriate to the aims of the study? |
|  | Were dietary factors and outcome variables measured correctly using instruments/ measurements that had been trialed, piloted or published previously? |
|  | Is it clear what was used to determine statistical significance and/or precision  estimates? (eg, p values, CIs) |
|  | Were the methods (including statistical methods) sufficiently described to enable them  to be repeated? |
|  | Were the basic data adequately described? |
|  | Were the results internally consistent? |
|  | Were the results for the analyses described in the methods presented? |
|  | Were the authors’ discussions and conclusions justified by the results? |
|  | Were the limitations of the study discussed? |
|  | Were there any funding sources or conflicts of interest that may affect the authors’ interpretation of the results? |
|  | Was ethical approval or consent of participants attained? |
| JBI Critical Appraisal Checklist for Case Control Studies | Were the groups comparable other than the presence of disease in cases or the absence of disease in controls? |
|  | Were cases and controls matched appropriately? |
|  | Were the same criteria used for identification of cases and controls? |
|  | Was exposure measured in a standard, valid and reliable way? |
|  | Was the exposure period of interest long enough to be meaningful? |
|  | Were outcomes assessed in a standard, valid and reliable way for cases and controls? |
|  | Was exposure measured in the same way for cases and controls? |
|  | Were confounding factors identified? |
|  | Were strategies to deal with confounding factors stated? |
|  | Was appropriate statistical analysis used? |
| Critical Appraisal Skills Programme (CASP) Cohort Study Checklist | Did the study address a clearly focused issue? |
|  | Was the follow up of subjects long enough? |
|  | Was the cohort recruited in an acceptable way? |
|  | Can the results be applied to the local population? |
|  | Was the exposure accurately measured to minimize bias? |
|  | Was the outcome accurately measured to minimize bias? |
|  | Have the authors identified all important confounding factors? |
|  | Have they taken account of the confounding factors in the design and/or analysis? |
|  | Was the follow up of subjects complete enough? |
|  | Do you believe the results? |
|  | Are results precise? |
|  | Do the results of this study fit with other available evidence? |

^a^ For studies focusing on First Nations populations in Canada, adherence to First Nations Governance Principles of OCAP® was assessed. While principles of OCAP® are specific to the Canadian context, community access, ownership, and control intellectual and cultural property were assessed for American studies using relevant criteria of the Aboriginal and Torres Strait Islander Quality Appraisal Tool.

^b^ For studies focusing on Inuit populations, adherence to principles of Inuit Qaujimajatuqangit was assessed.

**Table S4.** References included after full-text review.

| **References** |
| --- |
| Auger, N., Kofman, O., Kosatsky, T., & Armstrong, B. (2005). Low-level methylmercury exposure as a risk factor for neurologic abnormalities in adults. *Neurotoxicology*, *26*(2), 149-157. |
| Boucher, O., Burden, M. J., Muckle, G., Saint-Amour, D., Ayotte, P., Dewailly, E., Nelson, C., Jacobson, S. W., & Jacobson, J. L. (2011). Neurophysiologic and neurobehavioral evidence of beneficial effects of prenatal omega-3 fatty acid intake on memory function at school age. *The American journal of clinical nutrition*, *93*(5), 1025-1037. |
| Boucher, O., Burden, M. J., Muckle, G., Saint-Amour, D., Ayotte, P., Dewailly, É., Nelson, C., Jacobson, S. W., & Jacobson, J. L. (2012). Response inhibition and error monitoring during a visual go/no-go task in Inuit children exposed to lead, polychlorinated biphenyls, and methylmercury. *Environmental Health Perspectives*, *120*(4), 608-615. |
| Boucher, O., Muckle, G., Jacobson, J. L., Carter, R. C., Kaplan-Estrin, M., Ayotte, P., Dewailly, E., & Jacobson, S. W. (2014). Domain-specific effects of prenatal exposure to PCBs, mercury, and lead on infant cognition: results from the Environmental Contaminants and Child Development Study in Nunavik. *Environmental health perspectives*, *122*(3), 310-316. |
| Cartier, C., Muckle, G., Jacobson, S. W., Jacobson, J. L., Dewailly, E., Ayotte, P., Chevrier, C., & Saint-Amour, D. (2014). Prenatal and 5-year p, p′-DDE exposures are associated with altered sensory processing in school-aged children in Nunavik: a visual evoked potential study. *Neurotoxicology*, *44*, 8-16. |
| Després, C., Beuter, A., Richer, F., Poitras, K., Veilleux, A., Ayotte, P., Dewailly, E., Saint-Amour, D., & Muckle, G. (2005). Neuromotor functions in Inuit preschool children exposed to Pb, PCBs, and Hg. *Neurotoxicology and teratology*, *27*(2), 245-257. |
| Ethier, A. A., Muckle, G., Bastien, C., Dewailly, E., Ayotte, P., Arfken, C., Jacobson, S. W., Jacobson, J. L. & Saint-Amour, D. (2012). Effects of environmental contaminant exposure on visual brain development: a prospective electrophysiological study in school-aged children. *Neurotoxicology*, *33*(5), 1075-1085. |
| Grattan, L. M., Boushey, C., Tracy, K., Trainer, V. L., Roberts, S. M., Schluterman, N., & Morris Jr, J. G. (2016). The association between razor clam consumption and memory in the CoASTAL cohort. *Harmful Algae*, *57*, 20-25. |
| Jacobson, J. L., Jacobson, S. W., Muckle, G., Kaplan-Estrin, M., Ayotte, P., & Dewailly, E. (2008). Beneficial effects of a polyunsaturated fatty acid on infant development: evidence from the Inuit of Arctic Quebec. *The Journal of pediatrics*, *152*(3), 356-364. |
| Lamoureux-Tremblay, V., Chauret, M., Muckle, G., Maheu, F., Suffren, S., Jacobson, S. W., Jacobson, J. L., Ayotte, P., Lepore, F., & Saint-Amour, D. (2021). Altered functional activations of prefrontal brain areas during emotional processing of fear in Inuit adolescents exposed to environmental contaminants. *Neurotoxicology and teratology*, *85*, 106973. |
| McKeown-Eyssen, G. E., Ruedy, J., & Neims, A. (1983). Methyl mercury exposure in northern Quebec: II. Neurologic findings in children. *American Journal of Epidemiology*, *118*(4), 470-479. |
| Philibert, A., Fillion, M., Da Silva, J., Lena, T. S., & Mergler, D. (2022). Past mercury exposure and current symptoms of nervous system dysfunction in adults of a First Nation community (Canada). *Environmental Health*, *21*(1), 34. |
| Plusquellec, P., Muckle, G., Dewailly, E., Ayotte, P., Jacobson, S. W., & Jacobson, J. L. (2007). The relation of low-level prenatal lead exposure to behavioral indicators of attention in Inuit infants in Arctic Quebec. *Neurotoxicology and Teratology*, *29*(5), 527-537. |
| Saint-Amour, D., Roy, M. S., Bastien, C., Ayotte, P., Dewailly, E., Després, C., Gingras, S., & Muckle, G. (2006). Alterations of visual evoked potentials in preschool Inuit children exposed to methylmercury and polychlorinated biphenyls from a marine diet. *Neurotoxicology*, *27*(4), 567-578. |
| Takaoka, S., Fujino, T., Hotta, N., Ueda, K., Hanada, M., Tajiri, M., & Inoue, Y. (2014). Signs and symptoms of methylmercury contamination in a First Nations community in Northwestern Ontario, Canada. *Science of the Total Environment*, *468*, 950-957. |
| Tracy, K., Boushey, C. J., Roberts, S. M., Morris Jr, J. G., & Grattan, L. M. (2016). Communities advancing the studies of Tribal nations across their lifespan: Design, methods, and baseline of the CoASTAL cohort. *Harmful Algae*, *57*, 9-19. |

**Table S5.** References and reasons for exclusion for studies that were excluded after full-text review.

| **References** | **Primary Reason for Exclusion** |
| --- | --- |
| Akande, V. O., Hendriks, A. M., Ruiter, R. A., & Kremers, S. P. (2015). Determinants of dietary behavior and physical activity among Canadian Inuit: A systematic review. *International Journal of Behavioral Nutrition and Physical Activity, 12*(1). | No brain health outcome. |
| Anand, S. S., Tu, J. V., Awadalla, P., Black, S., Boileau, C., Busseuil, D., Desai, D., Després, J.-P., de Souza, R. J., Dummer, T., Jacquemont, S., Knoppers, B., Larose, E., Lear, S. A., Marcotte, F., Moody, A. R., Parker, L., Poirier, P., Robson, P. J., Smith, Eric E; Spinelli, John J; Tardif, J., Teo, K K., Tusevljak, N., Friedrich, M. G. (2016). Rationale, design, and methods for Canadian alliance for healthy hearts and minds cohort study (CAHHM) – A Pan Canadian cohort study. *BMC Public Health, 16*(1). | Does not focus on Indigenous people. |
| Anthony, R. (2013). Animistic pragmatism and native ways of knowing: Adaptive strategies for overcoming the struggle for food in the sub-Arctic. *International Journal of Circumpolar Health, 72*(1), 21224. | No brain health outcome. |
| Audet-Delage, Y., Ouellet, N., Dallaire, R., Dewailly, E., & Ayotte, P. (2013). Persistent organic pollutants and transthyretin-bound thyroxin in plasma of Inuit women of childbearing age. *Environmental science & technology, 47*(22), 13086-13092. | No brain health outcome. |
| Bélanger, M. C., Dewailly, É., Berthiaume, L., Noël, M., Bergeron, J., Mirault, M. É., & Julien, P. (2006). Dietary contaminants and oxidative stress in Inuit of Nunavik. *Metabolism, 55*(8), 989-995. | No brain health outcome. |
| Bélanger, M. C., Mirault, M. É., Dewailly, E., Berthiaume, L., & Julien, P. (2008). Environmental contaminants and redox status of coenzyme Q10 and vitamin E in Inuit from Nunavik. *Metabolism, 57*(7), 927-933. | No brain health outcome. |
| Blanchet, C., Dewailly, E., Ayotte, P., & Bruneau, S. (2000). Contribution of selected traditional and market foods to the diet of Nunavik Inuit women. *Canadian Journal of Dietetic Practice and Research, 61*(2), 50. | No brain health outcome. |
| Borré, K. (1991). Seal blood, Inuit blood, and diet: A biocultural model of physiology and cultural identity. *Medical anthropology quarterly, 5*(1), 48-62. | No brain health outcome. |
| Boucher, O., Jacobson, S. W., Plusquellec, P., Dewailly, É., Ayotte, P., Forget-Dubois, N., ... & Muckle, G. (2012). Prenatal methylmercury, postnatal lead exposure, and evidence of attention deficit/hyperactivity disorder among Inuit children in Arctic Quebec. *Environmental health perspectives, 120*(10), 1456-1461. | No brain health outcome. |
| Brown, R. A. (2016). Linoleic acid and alpha-linolenic acid have central roles in brain energy substrate provision, endogenous lipid production, immune and repair function, via peroxisomal beta-oxidation-related pathways? *Omega-3 Fatty Acids*, 413–428. | Does not focus on Indigenous people. |
| Budowski P. (1981). Review: nutritional effects of omega 3-polyunsaturated fatty acids. *Israel journal of medical sciences, 17*(4), 223–231 | Not a primary study. |
| Dallaire, R., Dewailly, E., Ayotte, P., Muckle, G., Laliberté, C., & Bruneau, S. (2008). Effects of prenatal exposure to organochlorines on thyroid hormone status in newborns from two remote coastal regions in Quebec, Canada. *Environmental Research, 108*(3), 387-392. | No brain health outcome. |
| Das U. N. (2000). Beneficial effect(s) of n-3 fatty acids in cardiovascular diseases: but, why and how?. *Prostaglandins, leukotrienes, and essential fatty acids, 63*(6), 351–362. | Does not focus on Indigenous people. |
| Donaldson, S. G., Van Oostdam, J., Tikhonov, C., Feeley, M., Armstrong, B., Ayotte, P., Boucher, O., Bowers, W., Chan, L., Dallaire, F., Dallaire, R., Dewailly, E., Edwards, J., Egeland, G. M., Fontaine, J., Furgal, C., Leech, T., Loring, E., Muckle, G., Nancarrow, T., Pereg, D., Plusquellec, P., Potyrala, M., Receveur, O., Shearer, R. G. (2010). Environmental contaminants and human health in the Canadian Arctic. *The Science of the total environment, 408*(22), 5165–5234. | Not a primary study. |
| Egeland, G. M., Berti, P., Soueida, R., Arbour, L. T., Receveur, O., & Kuhnlein, H. V. (2004). Age differences in vitamin A intake among Canadian Inuit. *Canadian Journal of Public Health, 95*, 465-469. | No brain health outcome. |
| El-Hayek, Y. H. (2007). Mercury contamination in Arctic Canada: Possible Implications for Aboriginal Health. *Journal on Developmental Disabilities, 13*(1), 67–89. | Not a primary study. |
| Fediuk, K., Hidiroglou, N., Madère, R., & Kuhnlein, H. V. (2002). Vitamin C in Inuit traditional food and women’s diets. *Journal of Food Composition and Analysis, 15*(3), 221–235. | No brain health outcome. |
| Fialkowski, M. K., McCrory, M. A., Roberts, S. M., Tracy, J. K., Grattan, L. M., & Boushey, C. J. (2010). Evaluation of dietary assessment tools used to assess the diet of adults participating in the Communities Advancing the Studies of Tribal Nations Across the Lifespan cohort. *Journal of the American Dietetic Association, 110*(1), 65–73. | No brain health outcome. |
| Golzadeh, N., Barst, B. D., Basu, N., Baker, J. M., Auger, J. C., & McKinney, M. A. (2020). Evaluating the concentrations of total mercury, methylmercury, selenium, and selenium:mercury molar ratios in traditional foods of the Bigstone Cree in Alberta, Canada. *Chemosphere, 250*, 126285. | No brain health outcome. |
| Gómez de Cedrón, M., Wagner, S., Reguero, M., Menéndez-Rey, A., & de Molina, A. R. (2020). Miracle Berry as a Potential Supplement in the Control of Metabolic Risk Factors in Cancer. *Antioxidants (Basel, Switzerland), 9*(12), 1282. | Does not focus on Indigenous people. |
| Hanning, R. M., Sandhu, R., MacMillan, A., Moss, L., Tsuji, L. J., & Nieboer, E. (2003). Impact on blood Pb levels of maternal and early infant feeding practices of First Nation Cree in the Mushkegowuk Territory of northern Ontario, Canada. *Journal of environmental monitoring : JEM, 5*(2), 241–245. | No brain health outcome. |
| Karagas, M. R., Choi, A. L., Oken, E., Horvat, M., Schoeny, R., Kamai, E., Cowell, W., Grandjean, P., & Korrick, S. (2012). Evidence on the human health effects of low-level methylmercury exposure. *Environmental health perspectives, 120*(6), 799–806. | Not a primary study. |
| Kekuda, T. P., Shree, V. D., Noorain, G. S., Sahana, B. K., & Raghavendra, H. L. (2019). Ethnobotanical uses, phytochemistry and pharmacological activities of Clerodendrum infortunatum L.(Lamiaceae): a review. *Journal of Drug Delivery and Therapeutics, 9*(2), 547-559. | Not a Canadian or U.S-based paper. |
| Kennedy, D. O., & Scholey, A. B. (2005). Sage and brain function. *Nutrition Abstracts and Reviews: Series A, 75*(8), 25N – 31N. | Not a primary study. |
| Krey, A., Ostertag, S. K., & Chan, H. M. (2015). Assessment of neurotoxic effects of mercury in beluga whales (Delphinapterus leucas), ringed seals (Pusa hispida), and polar bears (Ursus maritimus) from the Canadian Arctic. *The Science of the total environment, 509-510*, 237–247. | Does not focus on Indigenous people. |
| Kühn, T. (2014). Fish consumption and the risk of stroke. *Current Nutrition Reports, 3*(2), 94–101. | Not a primary study. |
| Kuhnlein, H. V., & Chan, H. M. (2000). Environment and contaminants in traditional food systems of northern indigenous peoples. *Annual review of nutrition, 20*, 595–626. | Not a primary study. |
| Kuhnlein, H. V., & Receveur, O. (1996). Dietary change and traditional food systems of indigenous peoples. *Annual review of nutrition, 16*, 417–442. | No brain health outcome. |
| Kuhnlein, H. V., & Receveur, O. (2007). Local cultural animal food contributes high levels of nutrients for Arctic Canadian Indigenous adults and children. *The Journal of nutrition, 137*(4), 1110–1114. | No brain health outcome. |
| Kuhnlein, H. V., Barthet, V., Farren, A., Falahi, E., Leggee, D., Receveur, O., & Berti, P. (2006). Vitamins A, D, and E in Canadian Arctic traditional food and adult diets. *Journal of food composition and analysis, 19*(6-7), 495-506. | No brain health outcome. |
| Kuhnlein, H. V., Chan, H. M., Leggee, D., & Barthet, V. (2002). Macronutrient, mineral and fatty acid composition of Canadian Arctic traditional food. *Journal of food composition and analysis, 15*(5), 545-566. | No brain health outcome. |
| Kuhnlein, H. V., Receveur, O., & Chan, H. M. (2001). Traditional food systems research with Canadian Indigenous Peoples. *International journal of circumpolar health, 60*(2), 112–122. | No brain health outcome. |
| Kuhnlein, H. V., Receveur, O., Soueida, R., & Berti, P. R. (2008). Unique patterns of dietary adequacy in three cultures of Canadian Arctic indigenous peoples. *Public Health Nutrition, 11*(4), 349-360. | No brain health outcome. |
| Kuhnlein, H. V., Receveur, O., Soueida, R., & Egeland, G. M. (2004). Arctic indigenous peoples experience the nutrition transition with changing dietary patterns and obesity. *The Journal of nutrition, 134*(6), 1447–1453. | No brain health outcome. |
| Kuhnlein, H.V., Kubow, S., & Soueida, R. (1991). Lipid components of traditional inuit foods and diets of Baffin Island. *Journal of Food Composition and Analysis, 4*, 227-236. | No brain health outcome. |
| Lee, E. T., Welty, T. K., Fabsitz, R., Cowan, L. D., Le, N. A., Oopik, A. J., Cucchiara, A. J., Savage, P. J., & Howard, B. V. (1990). The Strong Heart Study. A study of cardiovascular disease in American Indians: design and methods. *American journal of epidemiology, 132*(6), 1141–1155. | No brain health outcome. |
| Lemire, M., Kwan, M., Laouan-Sidi, A. E., Muckle, G., Pirkle, C., Ayotte, P., & Dewailly, E. (2015). Local country food sources of methylmercury, selenium and omega-3 fatty acids in Nunavik, Northern Quebec. *The Science of the total environment*, 509-510, 248–259. | No brain health outcome. |
| McGrath-Hanna, N. K., Greene, D. M., Tavernier, R. J., & Bult-Ito, A. (2003). Diet and mental health in the Arctic: is diet an important risk factor for mental health in circumpolar peoples?-a review. *International Journal of Circumpolar Health, 62*(3), 228-241. | Not a primary study. |
| Mead, E. L., Gittelsohn, J., Roache, C., Corriveau, A., & Sharma, S. (2013). A community-based, environmental chronic disease prevention intervention to improve healthy eating psychosocial factors and behaviors in indigenous populations in the Canadian Arctic. *Health education & behavior : the official publication of the Society for Public Health Education, 40*(5), 592–602. | No brain health outcome. |
| Muckle, G., Ayotte, P., Dewailly E, E., Jacobson, S. W., & Jacobson, J. L. (2001). Prenatal exposure of the northern Québec Inuit infants to environmental contaminants. *Environmental health perspectives, 109*(12), 1291-1299. | No brain health outcome. |
| Muckle, G., Dewailly, E., & Ayotte, P. (1998). Prenatal exposure of Canadian children to polychlorinated biphenyls and mercury. *Canadian journal of public health = Revue canadienne de sante publique, 89 Suppl 1*, S20–27. | No brain health outcome. |
| Muckle, G., Dewailly, E., Ayotte, P., Jacobson, S., & Jacobson, J. (2004). Contributions of PCBs, pesticides, MeHg and n-3 fatty acids to fetal growth and motor development in Inuit infants in Arctic Quebec . *NeuroToxicology, 25*(4), 672–672. | Unable to locate full article |
| Philibert, A., Fillion, M., & Mergler, D. (2020). Mercury exposure and premature mortality in the Grassy Narrows First Nation community: a retrospective longitudinal study. *The Lancet Planetary Health, 4*(4), e141-e148. | No brain health outcome. |
| Poulin, J., Gibb, H., Prüss-Üstün, A., & World Health Organization. (2008). *Mercury: assessing the environmental burden of disease at national and local levels*. | Does not focus on Indigenous people. |
| Receveur, O., Boulay, M., & Kuhnlein, H. V. (1997). Decreasing traditional food use affects diet quality for adult Dene/Metis in 16 communities of the Canadian Northwest Territories. *The Journal of nutrition, 127*(11), 2179-2186. | No brain health outcome. |
| Sabaratnam, V., Kah-Hui, W., Naidu, M., & David, P. R. (2013). Neuronal health–can culinary and medicinal mushrooms help?. *Journal of traditional and complementary medicine, 3*(1), 62-68. | Not a Canadian or U.S-based paper. |
| Spiegelaar, N., Martin, I. D., & Tsuji, L. J. (2019). Indigenous Subarctic Food Systems in Transition: Amino Acid Composition (Including Tryptophan) in Wild‐Harvested and Processed Meats. *International journal of food science, 2019*(1), 7096416. | No brain health outcome. |
| Stamler, C. J., Abdelouahab, N., Vanier, C., Mergler, D., & Chan, H. M. (2006). Relationship between platelet monoamine oxidase-B (MAO-B) activity and mercury exposure in fish consumers from the Lake St. Pierre region of Que., Canada. *Neurotoxicology, 27*(3), 429-436. | Does not focus on Indigenous people. |
| Wang, S. P., Yang, H., Wu, J. W., Gauthier, N., Fukao, T., & Mitchell, G. A. (2014). Metabolism as a tool for understanding human brain evolution: lipid energy metabolism as an example. *Journal of human evolution, 77*, 41-49. | Not a primary study. |
| Weihe, P., Hansen, J. C., Murata, K., Debes, F., Jørgensen, P. J., Steuerwald, U., ... & Grandjean, P. (2002). Neurobehavioral performance of Inuit children with increased prenatal exposure to methylmercury. *International Journal of Circumpolar Health, 61*(1), 41-49. | Not a Canadian or U.S-based paper. |
| Weisburger, J. H. (1988). Comparison of nutrition as customary in the Western World, the Orient, and northern populations (Eskimos) in relation to specific disease risks. *Arctic Medical Research, 47*, 110-120. | Not a primary study. |
| Wheatley, B., Paradis, S., Lassonde, M., Giguere, M. F., & Tanguay, S. (1997). Exposure patterns and long term sequelae on adults and children in two Canadian indigenous communities exposed to methylmercury. *Water, Air, and Soil Pollution, 97*, 63-73. | No brain health outcome. |
| Wheatley, M.A. (1997). Social and cultural impacts of mercury pollution on aboriginal peoples in Canada. *Water, Air, and Soil Pollution, 97*, 85-90. | No brain health outcome. |
| Whiting, S. J., & Mackenzie, M. L. (1998). Assessing the changing diet of indigenous peoples. *Nutrition reviews, 56*(8), 248-250. | No brain health outcome. |
| Yatsu, F. M. (1991). Strokes in Asians and Pacific-Islanders, Hispanics, and Native Americans. *Circulation, 83*(4), 1471-1472. | Does not focus on Indigenous people. |

**Table S6.** Scores for each criteria of the Aboriginal and Torres Strait Islander Quality Appraisal Tool.

| **Authors** | **Criteria/Questions *** | | | | | | | | | | | | | | |
| --- | --- | --- | --- | --- | --- | --- | --- | --- | --- | --- | --- | --- | --- | --- | --- |
|  | **1** | **2** | **3** | **4** | **5** | **6** | **7** | **8** | **9** | **10** | **11** | **12** | **13** | **14** | **Total (%)** |
| Boucher et al. (2011) | 0 | 0 | 0 | 0 | 0 | 0 | 0 | 0 | 0 | 0.5 | 0 | 0 | 0 | 0 | 3.6 |
| Boucher et al. (2012) | 0 | 0 | 0 | 0 | 0 | 0 | 0 | 0 | 0 | 0 | 0 | 0 | 0 | 0 | 0.0 |
| Boucher et al. (2014) | 0.5 | 0 | 0 | 0 | 0 | 0 | 0 | 0 | 0.5 | 0 | 0 | 0 | 0 | 0 | 7.1 |
| Cartier et al. (2014) | 0 | 0.5 | 0 | 0 | 0 | 0 | 0 | 0 | 0 | 0 | 0 | 0 | 0.5 | 0 | 7.1 |
| Després et al. (2005) | 0 | 0.5 | 0 | 0 | 0 | 0 | 0 | 0 | 0 | 0 | 0 | 0 | 0.5 | 0 | 7.1 |
| Ethier et al. (2012) | 0 | 0.5 | 0 | 0 | 0 | 0 | 0 | 0 | 0 | 0 | 0 | 0 | 0.5 | 0 | 7.1 |
| Grattan et al. (2016) | 0 | 0.5 | 0 | 0 | 0 | 0 | 0 | 0 | 0 | 0 | 0 | 0 | 0 | 0 | 3.6 |
| Jacobson et al. (2008) | 0 | 0 | 0 | 0 | 0 | 0 | 0 | 0 | 0 | 1 | 0 | 0 | 0 | 0 | 7.1 |
| Lamoureux-Tremblay et al. (2021) | 0 | 0.5 | 0 | 0 | 0 | 0 | 0 | 0 | 0 | 0 | 0 | 0 | 0 | 0 | 3.6 |
| Philibert et al. (2022) | 1 | 1 | 0 | 0 | NA | NA | NA | 1 | 0 | 0 | 0 | 0 | 0 | 0 | 27.3 |
| Saint- Amour et al. (2006) | 0 | 0.5 | 0 | 0 | 0 | 0 | 0 | 0 | 0 | 0 | 0 | 0 | 0.5 | 0 | 7.1 |
| Tracy et al. (2016) | 1 | 1 | 1 | 0 | 0 | 0 | 0 | 0 | 0.5 | 0 | 1 | 1 | 1 | 1 | 53.6 |
| McKeown-Eyssen & Ruedy (1983) | 0 | 0 | 0 | 0 | 0 | 0 | 0 | 0 | 0 | 0 | 0 | 0 | 1 | 0 | 7.1 |
| Takaoka et al. (2014) | 0 | 0.5 | 0 | 0 | 0 | 0 | 0 | 0 | 0 | 0 | 0 | 0 | 0 | 0 | 3.6 |
| Auger et al. (2005) | 0.5 | 0 | 0 | 0 | 0 | 0 | 0 | 0 | 0 | 0 | 0 | 0 | 0 | 0 | 3.6 |
| Plusquellec et al. (2007) | 0 | 0.5 | 0 | 0 | 0 | 0 | 0 | 0 | 0 | 0 | 0 | 0 | 0.5 | 0 | 7.1 |

**Note: 1. Did the research respond to a need or priority determined by the community?; 2. Was community consultation and engagement appropriately inclusive?; 3. Did the research have Indigenous research leadership?; 4. Did the research have Indigenous governance?; 5. Were local community protocols respected and followed?; 6. Did the researchers negotiate agreements in regards to rights of access to Indigenous Peoples’ existing intellectual and cultural property?; 7. Did the researchers negotiate agreements to protect Indigenous Peoples' ownership of intellectual and cultural property created through the research?; 8. Did Indigenous Peoples and communities have control over the collection and management of research materials?; 9. Was the research guided by an Indigenous research paradigm?; 10. Does the research take a strengths-based approach, acknowledging and moving beyond practices that have harmed Indigenous Peoples in the past?; 11. Did the researchers plan and translate the findings into sustainable changes in policy and/or practice?; 12. Did the research benefit the community?; 13. Did the research demonstrate capacity strengthening for Indigenous individuals?; 14. Did everyone involved in the research have opportunities to learn from each other?*

**Table S7.** Scores for each criteria of the Appraisal Tool for Cross-Sectional Studies (AXIS tool).

| **Authors** | **Criteria/Questions *** | | | | | | | | | | | | | | | | | | | | |
| --- | --- | --- | --- | --- | --- | --- | --- | --- | --- | --- | --- | --- | --- | --- | --- | --- | --- | --- | --- | --- | --- |
|  | **1** | **2** | **3** | **4** | **5** | **6** | **7** | **8** | **9** | **10** | **11** | **12** | **13** | **14** | **15** | **16** | **17** | **18** | **19** | **20** | **Total (%)** |
| Auger et al. (2005) | 1 | 1 | 1 | 1 | 0.5 | 0.5 | 0 | 1 | 1 | 0.5 | 0.5 | 1 | 1 | 1 | 0 | 1 | 1 | 1 | 1 | 1 | 80 |
| Plusquellec et al. (2007) | 1 | 1 | 0 | 1 | 1 | 1 | 1 | 1 | 1 | 1 | 1 | 1 | 1 | 1 | 0 | 1 | 0.5 | 1 | 1 | 1 | 87.5 |

**Note: 1. Were the aims/objectives of the study clear?; 2. Was the study design appropriate for the stated aim(s)?; 3. Was the sample size justified?; 4. Was the target/reference population clearly defined?; 5. Was the sample frame taken from an appropriate population base so that it closely represented the target/reference population under investigation?; 6. Was the selection process likely to select subjects/participants that were representative of the target/reference population under investigation?; 7. Were measures undertaken to address and categorize non-responders?; 8. Does the response rate raise concerns about non-response bias?; 9. If appropriate, was information about non-responders described?; 10. Were dietary factors and outcome variables measured appropriate to the aims of the study?; 11. Were dietary factors and outcome variables measured correctly using instruments/ measurements that had been trialed, piloted or published previously?; 12. Is it clear what was used to determine statistical significance and/or precision estimates? (e.g., p-values, CIs); 13. Were the methods (including statistical methods) sufficiently described to enable them to be repeated?; 14. Were the basic data adequately described?; 15. Were the results internally consistent?; 16. Were the results for the analyses described in the methods presented?; 17. Were the authors’ discussions and conclusions justified by the results?; 18. Were the limitations of the study discussed?; 19. Were there any funding sources or conflicts of interest that may affect the authors’ interpretation of the results?; 20. Was ethical approval or consent of participants attained?*

**Table S8.** Scores for each criteria of the JBI Critical Appraisal Checklist for Case Control Studies.

| **Authors** | **Criteria/Questions *** | | | | | | | | | | |
| --- | --- | --- | --- | --- | --- | --- | --- | --- | --- | --- | --- |
|  | **1** | **2** | **3** | **4** | **5** | **6** | **7** | **8** | **9** | **10** | **Total (%)** |
| McKeown-Eyssen & Ruedy (1983) | 0.5 | 1 | 1 | 0 | 0.5 | 0.5 | 1 | 1 | 1 | 1 | 75 |
| Takaoka et al. (2014) | 0 | 0 | 1 | 0.5 | 1 | 1 | 1 | 0 | 0 | 1 | 55 |

**Note: 1. Were the groups comparable other than the presence of disease in cases or the absence of disease in controls?; 2. Were cases and controls matched appropriately?; 3. Were the same criteria used for identification of cases and controls?; 4. Was exposure measured in a standard, valid and reliable way?; 5. Was the exposure period of interest long enough to be meaningful?; 6. Were outcomes assessed in a standard, valid and reliable way for cases and controls?; 7. Was exposure measured in the same way for cases and controls?; 8. Were confounding factors identified?; 9. Were strategies to deal with confounding factors stated?; 10. Was appropriate statistical analysis used?*

**Table S9.** Scores for each criteria of the *Critical Appraisal Skills Programme (CASP) Cohort Study Checklist.*

| **Authors** | **Criteria/Questions *** | | | | | | | | | | | | |
| --- | --- | --- | --- | --- | --- | --- | --- | --- | --- | --- | --- | --- | --- |
|  | **1** | **2** | **3** | **4** | **5** | **6** | **7** | **8** | **9** | **10** | **11** | **12** | **Total** |
| Boucher et al. (2011) | 1 | 1 | 1 | 1 | 1 | 1 | 1 | 1 | 1 | 1 | 1 | 1 | 100 |
| Boucher et al. (2012) | 1 | 1 | 1 | 1 | 1 | 1 | 1 | 1 | 1 | 1 | 1 | 1 | 100 |
| Boucher et al. (2014) | 1 | 0.5 | 1 | 1 | 1 | 1 | 1 | 0.5 | 0.5 | 1 | 1 | 1 | 87.5 |
| Cartier et al. (2014) | 1 | 1 | 1 | 1 | 1 | 1 | 1 | 1 | 1 | 1 | 1 | 1 | 100 |
| Després et al. (2005) | 1 | 1 | 1 | 1 | 1 | 1 | 1 | 1 | 1 | 1 | 1 | 1 | 100 |
| Ethier et al. (2012) | 1 | 1 | 1 | 0.5 | 0.5 | 1 | 1 | 1 | 1 | 1 | 1 | 1 | 91.7 |
| Grattan et al. (2016) | 1 | 1 | 1 | 1 | 1 | 1 | 1 | 1 | 1 | 1 | 1 | 1 | 100 |
| Jacobson et al. (2008) | 1 | 1 | 1 | 1 | 1 | 1 | 1 | 1 | 1 | 1 | 1 | 1 | 100 |
| Lamoureux-Tremblay et al. (2021) | 1 | 1 | 1 | 0.5 | 1 | 0.5 | 1 | 1 | 0.5 | 0.5 | 1 | 1 | 83.3 |
| Philibert et al. (2022) | 1 | 1 | NA | 1 | 0.5 | 0.5 | 1 | 1 | 0.5 | 1 | 1 | 1 | 86.4 |
| Saint- Amour et al. (2006) | 1 | 1 | 1 | 1 | 1 | 1 | 1 | 1 | 1 | 1 | 1 | 1 | 100 |
| Tracy et al. (2016) | 1 | NA | 1 | 1 | 1 | 1 | 1 | 1 | NA | 1 | 1 | 0.5 | 95 |

**Note: 1. Did the study address a clearly focused issue?; 2. Was the follow up of subjects long enough?; 3. Was the cohort recruited in an acceptable way?; 4. Can the results be applied to the local population?; 5. Was the exposure accurately measured to minimize bias?; 6. Was the outcome accurately measured to minimize bias?; 7. Have the authors identified all important confounding factors?; 8. Have they taken account of the confounding factors in the design and/or analysis?; 9. Was the follow up of subjects complete enough?; 10. Do you believe the results?; 11. Are results precise?; 12. Do the results of this study fit with other available evidence?*
